# Supplementary material for: Feeding Corn Silage or Grass Hay as Sole Dietary Forage Sources: Overall Mechanism of Forages Regulating Health-Promoting Fatty Acid Status in Milk of Dairy Cows
Source: Foods. 2023 Jan 9;12(2):303. doi: 10.3390/foods12020303 (PMC9857621; doi:10.3390/foods12020303)
Supplement: Supplementary file 1 [file foods-12-00303-s001.zip › foods-2122286-supplementary.pdf]

Table S1. Effects of different dietary forage sources on dominant fatty acids intake (g/d) and flows (g/d) across gastrointestinal tract (GIT) of cows.

Item	Diet [1]			SEM	Probability [2]		
	CS	MIX	GH		T	L	Q
Fatty acids intake							
C16:0	427.08	413.14	434.02	11.34	0.28	0.37	0.39
C18:0	26.66	28.35	32.31	1.14	0.21	0.13	0.78
C18:1 n-9c	145.23	127.24	115.78	9.30	0.37	0.06	0.35
C18:2 n-6c	275.11	236.03	201.95	10.60	0.02	0.03	0.64
C18:3 n-3	17.04	20.87	21.71	0.72	0.03	0.04	0.66
Total fatty acids	924.88	855.89	841.95	15.42	0.04	0.04	0.15
Duodenal fatty acids flow [3]							
C16:0	389.21	370.13	379.35	7.16	0.49	0.62	0.42
C18:0	396.80	339.14	314.56	15.02	0.09	0.02	0.47
C18:1 n-9c	49.73	47.28	42.97	1.51	0.07	0.08	0.75
C18:2 n-6c	40.14	37.48	35.79	1.56	0.38	0.32	0.89
C18:3 n-3	3.09	4.01	4.35	0.19	0.01	0.02	0.16
Total CLA	2.18	2.28	3.62	0.26	0.16	0.01	0.08
OCFA	13.84	10.75	10.68	0.64	0.07	0.02	0.15
HFA	402.27	380.96	390.21	7.52	0.48	0.56	0.40
Total fatty acids	902.66	825.49	795.04	16.24	0.02	< 0.01	0.11
Ileal fatty acids flow							
C16:0	80.81	53.47	44.09	6.06	0.02	0.01	0.17
C18:0	89.01	57.02	46.90	8.27	0.23	0.03	0.40
C18:1 n-9c	9.49	8.58	8.68	0.87	0.94	0.76	0.81
C18:2 n-6c	8.83	12.54	9.76	1.75	0.81	0.86	0.45
C18:3 n-3	1.68	1.54	1.12	0.12	0.02	0.06	0.57
Total CLA	0.10	0.10	0.14	0.02	0.74	0.28	0.54
OCFA	2.66	1.65	1.50	0.23	0.18	0.02	0.24
HFA	83.51	54.98	45.43	6.27	0.02	0.02	0.18
Total fatty acids	198.68	139.14	115.87	14.69	0.09	< 0.01	0.35
Rectal fatty acids flow							
C16:0	69.24	43.57	38.02	5.71	0.07	0.01	0.21
C18:0	75.97	47.53	39.23	6.40	0.14	0.01	0.21
C18:1 n-9c	7.93	7.04	5.91	0.44	0.44	0.07	0.92
C18:2 n-6c	6.90	6.32	5.58	0.41	0.50	0.25	0.96
C18:3 n-3	0.78	0.61	0.48	0.06	0.33	0.03	0.80
Total fatty acids	171.31	111.73	95.17	12.66	0.08	< 0.01	0.12

[1] CS = diet with 46% corn silage as the sole dietary forage source; MIX = diet with a mixture of 23% corn silage and 14% grass hays (6% alfalfa hay and 8% oat hay) as the dietary forage sources; GH = diet with 28% grass hays (12% alfalfa hay and 16% oat hay) as the sole forage source. [2] Probability of treatment effects: T = fixed effect of diet treatment; L = linear effect of corn silage proportions in diets; Q = quadratic effect of corn silage proportions in diets. Total CLA (conjugated linoleic acids) = CLA-c9t11 +CLA-t10c12, SUM C18= C18:0 +C18:1 n-9c + C18:2 n-6c + C18:3 n-3, OCFA = C13:0 + C15:0 +C17:0, HFA (hypercholesterolaemic FA) = C12:0 + C14:0 + C16:0.

Table S2. Effects of different dietary forage sources on ruminal biohydrogenation (%) of unsaturated C18 fatty acids of cows.

Item	Diet			SEM	Probability		
	CS	MIX	GH		T	L	Q
C18:1 n-9c	67.51	63.88	65.23	0.89	0.27	0.26	0.89
C18:2 n-6c	86.14	84.52	83.38	0.52	0.41	0.29	0.56
C18:3 n-3	82.78	81.37	81.26	1.42	0.37	0.57	0.46
Total C18	78.81	76.59	76.62	0.89	0.36	0.38	0.58

Table S3. Effects of different dietary forage sources on individual and grouped relative fatty acid composition (% of total fatty acids) in duodenal digesta of cows.

Item	Diet			SEM	Probability		
	CS	MIX	GH		T	L	Q
Total fatty acids [1], mg/g	86.86	88.07	94.01	0.77	0.44	0.21	0.44
Fatty acid composition							
C10:0	0.03	0.04	0.03	0.01	0.89	0.97	0.56
C12:0	0.33	0.26	0.31	0.02	0.42	0.66	0.21
C13:0	0.08	0.06	0.06	0.01	0.02	0.11	0.13
C14:0	1.09	1.04	1.03	0.06	0.90	0.74	0.91
C15:0	0.90	0.74	0.77	0.04	0.17	0.17	0.24
C16:0	42.31	44.42	46.73	1.01	0.19	0.09	0.99
C16:1	0.14	0.11	0.13	0.01	0.48	0.64	0.28
C17:0	0.53	0.49	0.49	0.01	0.27	0.28	0.57
C18:0	43.11	40.73	38.73	1.20	0.33	0.20	0.90
C18:1 n-9c	5.41	5.67	5.3	0.15	0.17	0.78	0.40
C18:2 n-6c	4.37	4.50	4.41	0.17	0.87	0.93	0.81
CLA-c9t11	0.18	0.21	0.36	0.03	0.06	0.02	0.18
CLA-t10c12	0.06	0.07	0.09	0.01	0.68	0.22	0.70
C18:3 n-3	0.34	0.48	0.54	0.02	0.09	0.06	0.26
C20:0	0.48	0.51	0.55	0.02	0.24	0.20	0.92
C20:1	0.08	0.09	0.09	0.01	0.24	0.44	0.68
C22:0	0.40	0.37	0.43	0.01	0.15	0.32	0.09
C22:1 n-9	0.09	0.15	0.11	0.01	0.06	0.27	0.01
C24:0	0.30	0.31	0.29	0.01	0.40	0.35	0.46
C24:1	0.02	0.02	0.01	0.01	0.93	0.79	0.64
Grouped fatty acids [2]							
MCFA	0.37	0.30	0.34	0.02	0.65	0.64	0.26
LCFA	99.63	99.7	99.66	0.02	0.65	0.64	0.26
SFA	89.54	88.97	89.52	0.34	0.45	0.99	0.50
MUFA	5.72	6.05	5.64	0.16	0.15	0.82	0.35
PUFA	4.74	4.98	4.84	0.19	0.73	0.85	0.68
Total C18	53.22	51.38	48.97	0.99	0.25	0.10	0.92
Total CLA	0.24	0.27	0.45	0.04	0.13	0.02	0.19
OCFA	1.51	1.29	1.32	0.06	0.16	0.17	0.30
HFA	43.73	45.72	48.07	1.02	0.22	0.10	0.93
PUFA n-3	0.34	0.48	0.54	0.02	0.09	0.06	0.26
PUFA n-6	4.37	4.50	4.41	0.17	0.87	0.93	0.81
n-6/n-3 ratio	13.06	9.32	8.25	0.51	0.06	0.08	0.27

[1] Total fatty acids concentration in dry digesta.[2] MCFA (medium-chain FA) = C10:0 + C12:0, LCFA (long-chain FA) = carbon chain > 12 (C13:0 to C24:1), SFA (saturated FA) = C10:0 + C12:0 + C13:0 + C14:0 + C15:0 + C16:0 + C17:0 + C18:0 + C20:0 + C22:0 + C24:0, MUFA (monounsaturated FA) = C16:1 + C18:1 n-9c + C20:1 + C22:1 n-9 + C24:1, PUFA (polyunsaturated FA) = C18:2 n-6c + C18:3 n-3, Total CLA (conjugated linoleic acids) = CLA-c9t11 +CLA-t10c12, SUM C18= C18:0 +C18:1 n-9c + C18:2 n-6c + C18:3 n-3, OCFA = C13:0 + C15:0 +C17:0, HFA (hypercholesterolaemic FA) = C12:0 + C14:0 + C16:0, PUFA n-3 = C18:3 n-3, PUFA n-6 = C18:2 n-6c, n-6/n-3 ratio = PUFA n-6/ PUFA n-3.

Table S4. Effects of different dietary forage sources on individual and grouped relative fatty acid composition (% of total fatty acids) in ileal digesta of cows.

Item	Diet			SEM	Probability		
	CS	MIX	GH		T	L	Q
Total fatty acids, mg/g	47.75	44.52	51.89	4.15	0.81	0.72	0.62
Fatty acid composition							
C10:0	0.04	0.03	0.03	0.01	0.44	0.34	0.45
C12:0	0.32	0.22	0.26	0.02	0.34	0.24	0.12
C13:0	0.05	0.04	0.05	0.01	0.33	0.47	0.30
C14:0	1.08	0.88	0.90	0.06	0.48	0.28	0.39
C15:0	0.79	0.66	0.78	0.03	0.27	0.93	0.10
C16:0	41.24	38.46	38.08	1.27	0.70	0.38	0.68
C16:1	0.11	0.12	0.12	0.01	0.86	0.47	0.87
C17:0	0.49	0.50	0.47	0.01	0.86	0.71	0.63
C18:0	44.09	41.37	40.54	1.62	0.79	0.45	0.80
C18:1 n-9c	4.77	6.13	7.44	0.68	0.54	0.15	0.96
C18:2 n-6c	4.52	8.56	8.39	1.17	0.57	0.21	0.39
CLA-c9t11	0.03	0.05	0.08	0.01	0.27	0.01	0.65
CLA-t10c12	0.01	0.02	0.03	0.004	0.41	0.06	0.80
C18:3 n-3	0.88	1.13	0.96	0.09	0.17	0.77	0.35
C20:0	0.53	0.66	0.64	0.03	0.33	0.16	0.22
C20:1	0.09	0.16	0.21	0.03	0.28	0.14	0.84
C22:0	0.39	0.43	0.46	0.02	0.08	0.05	0.84
C22:1 n-9	0.07	0.15	0.15	0.02	0.09	0.08	0.20
C24:0	0.45	0.45	0.43	0.01	0.83	0.59	0.63
C24:1	0.09	0.05	0.09	0.01	0.18	0.76	0.07
Grouped fatty acids							
MCFA	0.36	0.25	0.29	0.02	0.30	0.23	0.14
LCFA	99.64	99.75	99.71	0.02	0.30	0.23	0.14
SFA	89.47	83.7	82.64	1.70	0.56	0.12	0.47
MUFA	5.13	6.61	8.01	0.73	0.52	0.14	0.95
PUFA	5.41	9.70	9.35	1.15	0.55	0.19	0.33
sum C18	54.27	57.19	57.33	1.30	0.73	0.41	0.64
total CLA	0.05	0.07	0.11	0.01	0.28	0.01	0.67
OCFA	1.33	1.20	1.30	0.04	0.54	0.79	0.26
HFA	42.64	39.56	39.24	1.35	0.69	0.37	0.66
PUFA n-3	0.88	1.13	0.96	0.09	0.17	0.77	0.35
PUFA n-6	4.52	8.56	8.39	1.17	0.57	0.21	0.39
n-6/n-3 ratio	5.34	9.48	8.82	1.69	0.63	0.47	0.55

Table S5. Effects of different dietary forage sources on apparent digestibility (%) of individual and total fatty acids in the small intestine of cows.

Item	Diet			SEM	Probability		
	CS	MIX	GH		T	L	Q
Individual fatty acid							
C10:0	85.98	88.12	88.12	1.33	0.79	0.58	0.74
C12:0	79.28	85.57	87.15	1.69	0.08	0.06	0.42
C13:0	84.55	88.15	89.33	1.30	0.35	0.17	0.64
C14:0	78.53	85.78	87.27	1.70	0.03	0.03	0.28
C15:0	80.21	85.27	85.35	1.74	0.39	0.28	0.51
C16:0	79.17	85.37	88.38	1.59	0.02	0.01	0.42
C16:1	81.42	82.95	86.09	1.44	0.37	0.24	0.83
C17:0	79.55	83.22	86.06	1.53	0.33	0.11	0.86
C18:0	77.82	83.03	85.07	1.58	0.31	0.07	0.56
C18:1 n-9c	80.81	81.47	80.26	1.81	0.98	0.91	0.84
C18:2 n-6c	77.65	63.87	72.87	5.87	0.76	0.78	0.43
CLA-c9t11	95.94	95.96	96.96	0.57	0.82	0.37	0.85
CLA-t10c12	95.21	94.99	95.05	0.79	0.98	0.81	0.82
C18:3 n-3	55.67	61.72	74.35	4.60	0.01	0.01	0.65
C20:0	76.13	78.59	83.45	1.59	0.32	0.07	0.73
C20:1	74.69	70.85	68.57	3.11	0.67	0.50	0.91
C22:0	79.42	80.86	84.49	0.93	0.16	0.02	0.48
C22:1 n-9	83.64	83.36	81.48	1.75	0.90	0.67	0.86
C24:0	68.13	75.5	78.93	2.00	0.15	0.02	0.49
C24:1	43.27	67.08	68.16	4.26	0.03	0.01	0.01
Grouped fatty acids							
Total fatty acids	78.43	83.29	85.72	1.38	0.13	0.03	0.55
MCFA	79.36	86.14	87.26	1.56	0.12	0.03	0.25
LCFA	78.43	83.28	85.72	1.38	0.13	0.03	0.55
SFA	78.43	84.38	86.83	1.49	0.13	0.01	0.37
MUFA	80.54	81.3	80.01	1.79	0.98	0.92	0.82
PUFA	75.38	63.84	73.05	5.17	0.78	0.89	0.42
Total CLA	95.75	95.73	96.59	0.58	0.89	0.47	0.90
OCFA	80.22	84.62	85.83	1.61	0.37	0.20	0.65
HFA	79.16	85.38	88.36	1.59	0.02	0.01	0.46
PUFA n-3	45.67	61.72	74.35	4.60	0.01	0.01	0.65
PUFA n-6	77.65	63.87	72.87	5.87	0.76	0.78	0.43

Table S6. Effects of different dietary forage sources on relative fatty acid composition (% of total fatty acids) and concentrations of other metabolites (mM unless otherwise indicated) in arterial plasma of cows.

Item	Diet			SEM	Probability		
	CS	MIX	GH		T	L	Q
Total fatty acids, mg/100mL	313.46	298.35	293.49	11.79	0.91	0.89	0.55
Fatty acid composition							
C12:0	0.10	0.08	0.07	0.01	0.02	0.12	0.75
C13:0	0.20	0.24	0.20	0.01	0.07	0.93	0.13
C14:0	0.45	0.38	0.43	0.02	0.30	0.62	0.19
C15:0	0.28	0.28	0.18	0.03	0.15	0.15	0.34
C15:1	0.95	1.14	0.93	0.04	0.02	0.60	0.01
C16:0	14.60	15.07	14.48	0.16	0.36	0.77	0.16
C16:1	0.82	0.66	0.74	0.05	0.01	0.50	0.30
C17:0	0.77	1.14	0.79	0.07	0.19	0.87	0.01
C18:0	17.81	17.99	17.11	0.24	0.04	0.25	0.31
C18:1 n-9c	7.35	6.92	6.66	0.24	0.26	0.30	0.87
C18:2 n-6c	44.68	43.77	46.27	0.53	0.11	0.19	0.12
C18:3 n-3	2.24	2.58	3.01	0.14	0.07	0.01	0.81
C20:0	0.10	0.05	0.10	0.01	0.27	0.95	0.12
C21:0	0.17	0.15	0.13	0.01	0.25	0.02	0.91
C20:3 n-6	3.93	4.11	3.77	0.12	0.34	0.63	0.38
C20:4 n-6	2.66	2.49	2.43	0.12	0.02	0.50	0.87
C22:0	0.88	0.83	0.83	0.03	0.23	0.55	0.74
C20:5 n-3	0.30	0.30	0.26	0.02	0.59	0.54	0.62
C22:1 n-9	0.16	0.15	0.20	0.02	0.63	0.45	0.63
C23:0	0.74	0.80	0.68	0.02	0.01	0.11	0.01
C24:0	0.53	0.57	0.47	0.02	0.02	0.14	0.08
C24:1	0.27	0.28	0.24	0.01	0.04	0.23	0.19
Grouped fatty acids [1]							
MCFA	0.10	0.08	0.07	0.01	0.02	0.12	0.75
LCFA	99.90	99.92	99.93	0.01	0.02	0.12	0.75
SFA	36.63	37.58	35.49	0.44	0.09	0.26	0.10
MUFA	9.56	9.15	8.77	0.29	0.24	0.33	0.99
PUFA	53.81	53.26	55.75	0.54	0.11	0.13	0.16
OCFA	2.16	2.62	1.98	0.11	0.07	0.26	0.01
HFA	15.15	15.53	14.98	0.15	0.44	0.66	0.19
PUFA n-3	2.54	2.88	3.28	0.13	0.05	0.02	0.90
PUFA n-6	51.27	50.38	52.47	0.49	0.18	0.31	0.16
n-6/n-3 ratio	20.32	17.59	16.11	0.81	0.11	0.03	0.64
Other metabolites [2]							
Acetate	1.59	1.84	1.78	0.05	0.17	0.03	0.04
BHBA	0.79	0.84	0.83	0.02	0.89	0.67	0.70
VLDL	2.99	2.60	2.76	0.15	0.55	0.59	0.47
GLU	4.50	4.44	4.50	0.05	0.93	0.97	0.65
NEFA, umol/L	104.31	114.27	119.67	5.40	0.61	0.31	0.86

[1] MCFA (medium-chain FA) = C10:0 + C12:0, LCFA (long-chain FA) = carbon chain > 12 (C13:0 to C24:1), SFA (saturated FA) = C10:0 + C12:0 + C13:0 + C14:0 + C15:0 + C16:0 + C17:0 + C18:0 + C20:0 + C22:0 + C24:0, MUFA (monounsaturated FA) = C16:1 + C18:1 n-9c + C20:1 + C22:1 n-9 + C24:1, PUFA (polyunsaturated FA) = C18:3 n-3 + C20:5 n-3 + C18:2 n-6c + C20:3 n-6 + C20:4 n-6, Total CLA (conjugated linoleic acids) = CLA-c9t11 +CLA-t10c12, OCFA = C13:0 + C15:0 +C17:0, HFA (hypercholesterolaemic FA) = C12:0 + C14:0 + C16:0, PUFA n-3 = C18:3 n-3 + C20:5 n-3, PUFA n-6 = C18:2 n-6c + C20:3 n-6 + C20:4 n-6, n-6/n-3 ratio = PUFA n-6/ PUFA n-3. [2] BHBA = β-hydroxybutyric acid, VLDL = very low-density lipoprotein, GLU = glucose, NEFA = Nonestesterified fatty acid.

Table S7. Effects of different dietary forage sources on relative fatty acid composition (% of total fatty acids) and concentrations of other metabolites (mM unless otherwise indicated) in venous plasma of cows.

Item	Diet			SEM	Probability		
	CS	MIX	GH		T	L	Q
Total fatty acids, mg/100mL	258.31	250.52	244.14	14.96	0.82	0.91	0.50
Fatty acid composition							
C12:0	0.10	0.10	0.09	0.01	0.81	0.62	0.55
C13:0	0.20	0.21	0.19	0.01	0.93	0.74	0.63
C14:0	0.57	0.46	0.39	0.04	0.47	0.12	0.87
C15:0	0.33	0.30	0.29	0.04	0.89	0.76	0.94
C15:1	0.94	1.04	0.92	0.03	0.64	0.74	0.14
C16:0	15.23	14.96	14.48	0.20	0.64	0.17	0.82
C16:1	0.86	0.82	0.64	0.06	0.43	0.13	0.54
C17:0	0.76	0.93	0.82	0.05	0.44	0.64	0.18
C18:0	17.59	16.96	17.14	0.25	0.58	0.51	0.50
C18:1 n-9c	7.23	7.30	6.25	0.22	0.23	0.04	0.14
C18:2 n-6c	44.10	44.66	46.85	0.57	0.16	0.04	0.42
C18:3 n-3	2.37	2.82	2.48	0.09	0.12	0.53	0.04
C20:0	0.06	0.07	0.07	0.01	0.54	0.36	0.83
C21:0	0.17	0.19	0.16	0.01	0.72	0.77	0.27
C20:3 n-6	4.01	3.82	3.95	0.13	0.91	0.87	0.65
C20:4 n-6	2.72	2.30	2.51	0.12	0.57	0.50	0.26
C22:0	0.86	1.02	0.88	0.08	0.74	0.92	0.44
C20:5 n-3	0.31	0.24	0.29	0.02	0.57	0.80	0.34
C22:1 n-9	0.15	0.17	0.16	0.01	0.86	0.79	0.63
C23:0	0.72	0.77	0.71	0.01	0.13	0.72	0.01
C24:0	0.50	0.56	0.49	0.01	0.27	0.84	0.05
C24:1	0.25	0.29	0.26	0.01	0.03	0.51	0.03
Grouped fatty acids							
MCFA	0.10	0.10	0.09	0.01	0.81	0.62	0.55
LCFA	99.90	99.90	99.91	0.01	0.81	0.62	0.55
SFA	37.08	36.54	35.70	0.47	0.61	0.30	0.89
MUFA	9.43	9.61	8.22	0.27	0.17	0.03	0.08
PUFA	53.5	53.85	56.07	0.59	0.32	0.08	0.40
OCFA	2.17	2.41	2.16	0.06	0.48	0.95	0.09
HFA	15.9	15.52	14.96	0.24	0.61	0.15	0.86
PUFA n-3	2.67	3.06	2.77	0.08	0.08	0.52	0.03
PUFA n-6	50.82	50.79	53.30	0.60	0.28	0.09	0.28
n-6/n-3 ratio	19.10	16.61	19.28	0.59	0.01	0.89	0.04
Other metabolites							
acetate	0.64	0.68	0.66	0.01	0.70	0.55	0.42
BHBA	0.52	0.55	0.55	0.01	0.12	0.15	0.60
VLDL	2.18	1.93	2.03	0.09	0.29	0.53	0.40
GLU	3.74	3.69	3.74	0.10	0.98	0.98	0.84
NEFA, umol/L	134.12	146.92	149.87	6.44	0.68	0.39	0.75

Table S8. Effects of different dietary forage sources on relative fatty acid composition (% of total fatty acids) in milk of cows.

Item	Diet			SEM	Probability		
	CS	MIX	GH		T	L	Q
Total fatty acids, mg/mL	43.26	42.78	44.66	1.629	0.42	0.73	0.32
Fatty acid composition							
C4:0	2.98	3.15	3.21	0.036	0.07	0.01	0.139
C6:0	1.20	1.25	1.24	0.040	0.34	0.71	0.74
C8:0	0.85	0.93	0.95	0.042	0.13	0.42	0.71
C10:0	2.19	2.61	2.61	0.152	0.02	0.03	0.14
C12:0	2.90	3.62	3.57	0.212	0.01	0.23	0.40
C13:0	0.11	0.14	0.13	0.012	0.56	0.55	0.46
C14:0	9.46	10.93	10.12	0.351	0.05	0.62	0.13
C14:1	0.89	1.11	1.06	0.044	0.07	0.01	0.11
C15:0	0.92	1.07	0.95	0.066	0.68	0.88	0.42
C16:0	36.78	37.68	36.51	0.642	0.76	0.35	0.81
C16:1	1.98	2.26	2.32	0.112	0.09	0.06	0.36
C17:0	0.48	0.44	0.44	0.017	0.06	0.39	0.68
C18:0	10.44	8.70	8.88	0.342	0.02	0.31	0.04
C18:1 n-9c	24.33	21.58	23.12	1.006	0.43	0.94	0.51
C18:2 n-6c	2.79	2.91	3.15	0.074	0.18	0.05	0.66
C18:3 n-3	0.32	0.37	0.42	0.015	0.02	0.01	0.70
CLA-c9t11	0.16	0.18	0.21	0.006	0.06	0.01	0.88
CLA-t10c12	0.01	0.01	0.02	0.001	0.07	0.01	0.12
C20:0	0.15	0.13	0.14	0.008	0.42	0.64	0.30
C20:1	0.09	0.07	0.08	0.005	0.02	0.74	0.27
C20:2	0.05	0.03	0.05	0.004	0.29	0.91	0.07
C20:3 n-3	0.02	0.01	0.02	0.003	0.31	0.93	0.07
C20:3 n-6	0.27	0.21	0.25	0.015	0.23	0.6	0.19
C20:4 n-6	0.25	0.22	0.25	0.009	0.20	0.93	0.21
C20:5 n-3	0.03	0.03	0.04	0.001	0.15	0.05	0.31
C21:0	0.06	0.05	0.07	0.005	0.18	0.74	0.04
C22:0	0.07	0.07	0.07	0.002	0.98	0.92	0.93
C22:1 n-9	0.29	0.34	0.27	0.018	0.14	0.54	0.15
C22:2	0.01	0.01	0.01	0.001	0.25	0.57	0.19
C23:0	0.04	0.04	0.04	0.001	0.72	0.51	0.82
C24:0	0.04	0.04	0.04	0.002	0.79	0.48	0.98
C24:1	0.01	0.01	0.01	0.001	0.20	0.82	0.50
Health Indices [1]							
Σ HFA	49.13	52.23	50.21	1.105	0.08	0.72	0.34
Σ PUFA n-3	0.37	0.41	0.47	0.013	0.04	0.01	0.37
Σ PUFA n-6	3.31	3.34	3.65	0.084	0.22	0.08	0.39
n-6/n-3 ratio	8.90	8.17	7.68	0.239	0.04	0.02	0.57

[1] Σ HFA (hypercholesterolaemic FA) = C12:0 + C14:0 + C16:0; Σ PUFA n-3 = C18:3 n-3 + C20:3 n-3 + C20:5 n-3; Σ PUFA n-6 = C18:2 n-6c + C20:3 n-6 + C20:4 n-6; n-6/n-3 ratio = Σ PUFA n-6/ Σ PUFA n-3.
